# Outcomes of patients with Down syndrome and acute leukemia: A retrospective observational study

**DOI:** 10.1097/MD.0000000000027459

**Published:** 2021-10-08

**Authors:** Madalina-Petronela Schmidt, Anca Colita, Anca-Viorica Ivanov, Daniel Coriu, Ingrith-Crenguta Miron

**Affiliations:** a “Sf Maria” Children's Hospital-Hemato-Oncology Department, Iasi, Romania; b Fundeni Clinical Institut - Pediatrics Department, Bucharest, Romania; c “Carol Davila” University of Medicine and Pharmacy, Bucharest, Romania; d “Grigore T Popa” University of Medicine and Pharmacy, Iasi, Romania; e Fundeni Clinical Institut-Hematology Department, Bucharest, Romania.

**Keywords:** acute leukemia, Down syndrome, outcomes, survival

## Abstract

Children with Down syndrome (DS) have a higher risk of developing acute leukemia than do those without DS. There are few studies in the literature about outcome, survival, and difficulties of treating patients with DS and acute leukemia in a developing country. This study aimed to analyze the outcome, response to treatment, survival, treatment complications, and causes of death in patients with DS and acute leukemia compared with those in patients with acute leukemia without DS diagnosed in the same period of time.

We conducted a retrospective observational analysis including a cohort of 21 patients with DS and acute leukemia diagnosed between 2009 and 2018 in 3 hemato-oncology centers (2 pediatric centers and 1 adult hematology center). A group of patients with DS-acute lymphoblastic leukemia (DS-ALL) was analyzed and compared with a group of 165 patients with acute lymphoblastic leukemia without DS, and a group of patients with DS-acute myeloid leukemia (DS-AML) was analyzed and compared with a group of 50 patients with acute myeloid leukemia without DS, which was diagnosed during the same period of time (2009–2018) and treated under similar conditions in terms of both treatment protocols and economic resources.

The overall survival rates in children with DS-ALL and DS-AML were 35.7% and 57.1%, respectively (*P* = .438). The overall survival rate was significantly worse in children with DS-ALL than in those with acute lymphoblastic leukemia without DS (35.71% vs 75.80%, *P* = .001). We noted that treatment-related mortality in the patients with DS-ALL was high (50%) (infections and toxicities related to chemotherapy); this result was significantly different from that for patients with leukemia without DS (*P* < .0001). The relapse rate was higher in patients with DS-ALL but not significantly higher than that in patients without DS (*P* = .13).

In contrast, the overall survival rate was better for patients with DS-AML than for those with acute myeloid leukemia without DS (57.1% vs 45.1%, *P* = .47).

Because of the particularities of the host, we suggest that DS-ALL and DS-AML should be considered as independent diseases and treated according to specific protocols with therapy optimization per the minimal residual disease.

## 1 Introduction

Children with Down syndrome (DS) have a higher risk of developing acute leukemia, including both acute myeloid leukemia (AML) and acute lymphoblastic leukemia (ALL), than do children without DS.^[1]^

AML in patients with DS (DS-AML) is associated with several characteristic features, one of which is a higher prevalence of acute megakaryoblastic leukemia (AMKL). The risk of developing AMKL is 500-fold higher for children with DS than for those without DS. DS-AMKL is characterized by a multistep transformation; in most cases, it is preceded by transient myeloproliferative disorder (TMD), a clonal myeloproliferative syndrome that occurs in the fetus or in the first few days after birth.^[1]^

TMD is identified in approximately 10% newborns with DS, and it is defined by the presence of megakaryoblasts in the peripheral blood. TMD resolves spontaneously in most cases (80%), usually in the first 3 months of life. In 20% to 30% cases with a history of TDM, transformation into AMKL is observed until the age of 4 years.^[2]^

Mutations in *GATA1*, the gene that encodes an essential hematopoietic transcription factor, occur in almost all patients with concomitant DS and TMD or AMKL. The presence of *GATA1* mutations link the 2 entities (TDM and AMKL) from a clonal perspective.^[3]^

Roberts et al^[3]^ evaluated a group of 200 newborns with DS and showed that up to 30% newborns with DS have *GATA1* mutations detected by conventional methods or next generation sequencing. The subgroup of patients in whom *GATA1* mutations could be detected only by next generation sequencing were diagnosed with “silent TDM,” which affects up to 20% newborns with DS.^[3]^

Trisomy 21 is the first step in the development of AMKL, followed by the addition of *GATA 1* mutations, which leads to TDM. The third step is the development of AMKL from a previous TDM clone that acquires mutations in the cohesin components (53%), CCCTC- binding factor (20%), or other epigenetic regulatory factors (EZH2, KANSL1).^[4]^

Before receiving a diagnosis of AML, patients with DS develop myelodysplasia, which is associated with progressive anemia, thrombocytopenia, and the presence of erythroblasts or megakaryocytes with dysplastic morphologies in the bone marrow.^[2]^

The clinical outcomes of patients with DS-AML are better than those of children with AML without DS, as well as DS alone without AML; such patients represent a subgroup with a favorable prognosis.^[1]^ Studies have shown that increasing the doses of chemotherapy to the levels used in children without DS leads to an increase in the number of deaths due to infections and cardiotoxicity^[5–7]^; therefore, it is very important to select a dose that is high enough to ensure efficacious therapy, but low enough to minimize treatment-related toxicity. It is worth noting that the unique sensitivity of myeloblasts to chemotherapy, including cytarabine, in patients with DS can be explained by the generation of increased intracellular levels of the metabolite cytosine arabinoside, cytarabine (ARA-C) triphosphate.^[8–11]^ This sensitivity explains the high efficacy of high-dose cytarabine (HD-ARA-C) cycles in improving treatment outcomes.

The incidence of ALL is 10 times higher in children with DS than in those without it.^[12,13]^ Patients with ALL and DS (DS-ALL) also exhibit characteristic clinical and biological features. DS-ALL is extremely rare in those under the age of 1 year, and the incidence peaks at a slightly older age than that in children with ALL without DS, including adolescents and young adults.^[14]^

Another peculiarity is the absence of the T-cell phenotype. In terms of gene expression and cytogenetics, patients with DS-ALL exhibit a lower incidence of chromosomal rearrangements, resulting in a favorable prognosis (ETV6-RUNX1, hyperdiploidy), as well as a lower likelihood of an unfavorable prognosis (BCR-ABLl, AF4-MLL).^[14]^ An abnormal expression of the CRLF2 cytokine receptor (a receptor expressed on Th2 cells, macrophages, dendritic cells) was identified in approximately 60% of all DS-ALL cases,^[15]^ and with the exception of trisomy 21, the most common cytogenetic abnormality is extrachromosomal X, which is observed in approximately 50% patients.^[16]^ The prognosis of DS-ALL patients is worse than that of children with ALL without DS due to both a higher mortality rate related to chemotherapy-induced toxicity and a higher frequency of relapses.^[17]^

Clinical trials tend to exclude patients with DS, so we consider it is useful to analyze a cohort of this specific subgroup of patients with acute leukemia and DS, diagnosed and treated in a developing country. There are few studies in the literature on the outcome, survival, and difficulties of treating patients with DS and acute leukemia in a developing country as it is a rare condition and the number of patients is few. A previous retrospective study explored some clinical and laboratory features in a group of patients from Romania who had DS and acute leukemia or transient leukemia.^[18]^

Our study analyzes the survival, outcome, treatment-related mortality (TRM), treatment difficulties, and complications, and performs a comparative analysis of patients with DS and acute leukemia with patients with ALL and those with AML diagnosed and treated in similar conditions. The primary endpoint was to determine and analyze differences in survival and outcome between DS-ALL and DS-AML patients, DS-ALL and ALL patients, and DS-AML and AML patients. We introduced patients diagnosed in an adult hematology center to the group of patients with DS and acute leukemia; we consider this a novelty because the diagnosis of acute leukemia in patients with DS in adulthood is rare.

## 2 Materials and methods

### 2.1 Patients

We conducted a retrospective analysis of a cohort of patients with DS-ALL and DS-AML diagnosed between 2009 and 2018 at 3 main hemato-oncology centers in Romania, 2 of which were pediatric centers (“Fundeni” Clinical Institute Bucharest-Pediatrics Clinic and “Sf. Maria” Children's Hospital Iasi, Hemato-Oncology Department), and one of which was an adult hematology center (“Fundeni” Clinical Institute Bucharest, Hematology Department). We included patients with complete data at the time of collection in the analysis.

The inclusion criteria for the group of DS patients included a confirmed cytogenetic diagnosis of DS (trisomy 21 or mosaicism) and a diagnosis of 1 of the 2 types of acute leukemia (ALL or AML) according to the World Health Organization criteria.^[19]^ Infants under 3 months of age were included in the study if the number of the blasts in their bone marrow exceeded 30%. Accordingly, 24 patients with DS and acute leukemia were examined for eligibility, and eventually, 21 patients with completed assessments and complete data were included in the study. There were 7 patients with DS-AML and 14 with DS-ALL. Three of the patients were diagnosed in the adult center and the other 18 patients were from the 2 pediatric centers.

The group of patients with DS-ALL was compared with a group of 165 ALL patients without DS and the group of patients with DS-AML was compared with a group of 50 AML patients without DS, both diagnosed between 2009 and 2018 at “Sf. Maria” Children's Hospital Iasi. The inclusion criteria for the group of patients with ALL and patients with AML were the diagnosis of ALL and AML, respectively, according to the World Health Organization criteria,^[19]^ absence of DS, and administration of the induction treatment in the center where they were diagnosed, that is, “Sf. Maria” Children's Hospital Iasi.

We examined 177 patients with ALL and 55 patients with AML for eligibility and included 165 patients with ALL without DS and 50 patients with AML without DS, who completed all assessments.

The study was approved by the Ethics Committees of the “Sf. Maria” Children's Hospital Iasi (6877/26.02.2020). Informed consent was obtained from the guardians of each patient. Only patients with complete follow-up data were included. Patients were followed up until death or until June 2019, whichever was earlier.

The demographic characteristics of all patients were recorded, as well as the presence of associated diseases, clinical features, hematological values at onset, the presence of blasts in the bone marrow aspirates, the presence of myelodysplastic elements at onset, immunophenotypic and cytogenetic examination data, molecular biological abnormalities, response to treatment on day 33 for ALL patients and on day 28 for AML patients, minimal residual disease (MRD), type of treatment, toxicity of therapy, cause of death, mortality during induction, remission rates, relapse rates, and survival. The first author checked and corrected the coding of categorical variables and incorrect values of continuous variables.

### 2.2 Treatment and monitoring

The 7 DS-AML patients were treated according to the standard AML protocols (4 patients were treated with the AIEOP LAM 2002 protocol,^[20]^ 2 with the BFM-AML 2004 protocol,^[21]^ and 1 with low-dose cytarabine).

The AIEOP LAM 2002 protocol (reduced intensity arm for children with AML-DS) primarily differs from the BFM-AML 2004 protocol in the following aspects: lower cumulative dose of etoposide (200 mg/m^2^ vs 950 mg/m^2^); higher cumulative number of days of continuous intravenous administration of cytarabine (12 days vs 2 days), albeit with a lower cumulative total dose of cytarabine (12,400 mg/m^2^ vs 29,400 mg/m^2^); and the lack of maintenance therapy.

The ML DS 2006 protocol was not used for any patient.^[22]^

The 14 DS-ALL patients were treated according to 3 different standard ALL protocols (11 patients with the ALL-IC-BFM 2002,^[23]^ 1 with the GMALL 07/2003,^[24]^ and 2 patients with the FRALLE 2000^[25]^).

The GMALL 07/2003 protocol is used for the treatment of ALL in adults and primarily differs from pediatric protocols (ALL-IC-BFM 2002 and FRALLE 2000) in the following aspects: lower cumulative dose of methotrexate and lower number of asparaginase doses in the induction and reinduction phases.

Patients with ALL without DS were treated according to the ALL-IC-BFM 2002 protocol^[23]^ and patients with AML without DS were treated according to the AIEOP LAM 2002 protocol.^[20]^

The response to treatment was assessed according to the criteria of the International Working Group.^[26]^ Complete remission was defined as <5% blasts in bone marrow aspirate smears, normal erythropoiesis, granulopoiesis and megakaryocytopoiesis, absolute neutrophil count >1 × 10^9^/L, and platelets >100 × 10^9^/L, absence of blasts in the cerebrospinal fluid or elsewhere.

After 2017, the response to treatment was also evaluated based on MRD with flow cytometry detection.^[27]^ Immunophenotyping was carried out using a FACS Canto II Flow Cytomer (BD Biosciences San Jose, CA).

In the specific case of an extramedullary relapse at the lower eyelid, a histopathological examination with an immunohistochemical analysis was performed.

### 2.3 Genetic analyses

A cytogenetic analysis was carried out using the G banding technique.^[28]^ Reverse-transcription polymerase chain reaction (RT-PCR) analysis was used to screen for the following gene fusions: BCR-ABL1, ETV6/RUNX1 (TEL-AML1), MLL-AF4, E2A-PBX, SIL-TAL, NPM1, FLT3-ITD, PML-RARα, CBFβ-MYH11, and AML1-ETO. These fusion genes were a part of the usual molecular diagnostic panel for patients with acute leukemia, diagnosed in our centers.

### 2.4 Statistical analysis

All statistical analyses were performed using IBM SPSS, version 25.0 (Armonk, NY).^[29]^ Descriptive analysis was performed using percentages and frequencies for categorical variables and medians with maximum and minimum values for continuous quantitative variables. Overall survival (OS) was measured from diagnosis to death or the last follow-up. Early death was defined as death due to any cause within the first 6 weeks after the disease onset. The Kaplan–Meier method was used to estimate the OS and the subgroups were compared using log-rank tests. *P*-values were derived from 2-sided tests and were considered significant if <.05. Bivariate analysis was used to analyze the relationship between the observed variables. The median follow-up time was 3.5 years (14 days–10.5 years) for DS-AML, 3 years (2 months–6.5 years) for DS-ALL, 5.3 years (1 month–11.6 years) for non-DS-ALL, and 4.5 years (14 days–10.6 years) for non-DS-AML.

## 3 Results

### 3.1 DS-ALL versus ALL patients

Table 1 shows the comparative analysis of DS-ALL patients with a large group of non-DS-ALL patients (n = 165) diagnosed during the same period in “Sf. Maria” Children's Hospital Iasi.

**Table 1:** Clinical and biological features of patients with DS-ALL and ALL patients without DS.

	DS-ALL (n = 14)	ALL (n = 165) without DS	*P*
Median age of diagnosis, yrs	5.3	5.2	.915
Sex: male/female	35.7%/64.2%	66.2%/33.7%	.02
Median WBC count (range)	5100/mm^3^ (380–144,490)	13,240/mm^3^ (420–1,000,000)	.281
Median Hb concentration (range)	7.5 g/dL (4.8–13)	6.8 g/dL (2.4–13.4)	.218
Median Plt count (range)	25,000/mm^3^ (8000–246,000)	38,000/mm^3^ (4000–573,000)	.494
Congenital heart disease			
Yes	5 (35.7%)	0 (0%)	
No	9 (64.2%)	165 (100%)	
CNS infiltration			.294
Yes	0 (0%)	12 (7.2%)	
No	14 (100%)	153 (92.7%)	
Molecular abnormalities (BCR-ABL1, TEL-AML1, MLL-AF4, E2A-PBX)	0 (0%)	38 (23%)	.048
BCR-ABL1	0 (0%)	3 (1.81%)	
TEL-AML1	0 (0%)	25 (15.15%)	
MLL-AF4	0 (0%)	3 (1.81%)	
E2A-PBX	0 (0%)	7 (4.24%)	

ALL = acute lymphoblastic leukemia, CNS = central nervous system, DS = Down syndrome, DS-ALL = Down syndrome with acute lymphoblastic leukemia, Hb = hemoglobin, Plt = platelet, WBC = white blood cell.

It is important to note the higher female:male ratio (1.8:1), with a predominance of female DS-ALL patients (*P* = .02), as well as the fact that patients with DS did not display molecular abnormalities based on the RT-PCR assessments (*P* = .048). Only one DS-ALL patient presented hyperdiploidy.

In the DS-ALL group, the median age at the time of diagnosis was 5.3 years. There were no cases diagnosed in infants. The median white blood cell (WBC) count at diagnosis was 5100/mm^3^ (range: 380–144,490), the median hemogoblin level was 7.5 g/dL (range: 4.8–13), and the median platelet count was 25,000/mm^3^ (8000–246,000). Regarding the percentages of blasts, the median value in the peripheral blood was 27% while in the bone marrow, it was 82%. According to bivariate regression analysis, there were no statistically significant differences between the DS-ALL and non-DS-ALL groups regarding age and hematological values at onset (Table 1).

The fragility of patients with DS-ALL can be seen from the description of the associated diseases, which influences the toxicity related to the treatment. Five (35%) patients with DS-ALL had associated congenital heart disease (atrial septal defect, ventricular septal defect, interventricular septal aneurysm, persistent common atrioventricular canal, coronary artery disease, and Teratology of Fallot). Three of the patients underwent heart surgery. Five patients exhibited severe mental retardation. Other congenital malformations included congenital cataracts, brachydactyly, congenital limb defects, and strabismus. None of the DS-ALL patients experienced central nervous system (CNS) infiltration or T-cell acute lymphoblastic leukemia (T-ALL), while in the group without DS, 7.2% had CNS infiltration at onset (*P* = .29) and 15.7% had T-ALL. Hepatomegaly and splenomegaly were predominant at the onset (in 50% and 64% cases, respectively). Cytogenetic examination revealed only trisomy 21 in 10 (71.4%) patients, while 2 patients of the DS-ALL group had other associated abnormalities (monosomy 8, Robertsonian translocation t(14q;21q); 1 patient had mosaicism and another had 56 hyperdiploid chromosomes).

Table 2 highlights the comparative analysis of treatment response, treatment-related toxicity, and OS between DS-ALL and non-DS-ALL patients.

**Table 2:** Outcome of patients with DS-ALL and ALL without DS.

	DS-ALL (n = 14)	ALL without DS (n = 165)	*P*
Response after induction	12 (85.7%)	151 (91.5%)	.093
CR	11 (78.5%)	147 (89%)	
Poor prednisone response	3 (21.4%)	23 (13.9%)	.467
Relapse	4 (28.5%)	22 (13.3%)	.13
Early relapse	3 (21.4%)	18 (10.9%)	
Late relapse	1 (7.1%)	4 (2.42%)	
Bone marrow relapse	3 (21.4%)	16 (9.69%)	
CNS relapse	1 (7.1%)	6 (3.63%)	
Extramedullary	0 (0%)	2 (1.21%)	
Treatment-related mortality	7 (50%)	24 (14.5%)	<.0001
Overall survival	35.7%	75.8%	.001
Median follow-up time	3 years (2 months–6.5 years)	5.3 years (1 month–11.6 years)	

ALL = acute lymphoblastic leukemia, CNS = central nervous system, CR = complete remission, DS = Down syndrome, DS-ALL = Down syndrome with acute lymphoblastic leukemia, PR = partial remission.

**Figure 1:**
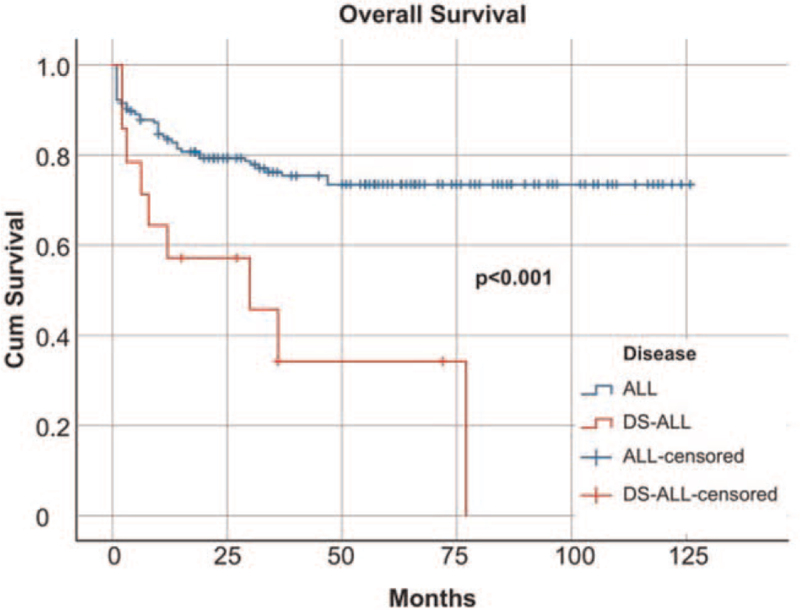
Overall survival in ALL without DS (blue line) and DS-ALL (red line). ALL = acute lymphoblastic leukemia, DS = Down syndrome, DS-ALL = Down syndrome-acute lymphoblastic leukemia.

The outcomes in patients with DS-ALL were unfavorable. Although 78.5% patients achieved complete remission after induction, the OS was only 35.7%, which was statistically significantly lower than the OS of patients with ALL without DS (75.8%; odds ratio, 2.12; 95% confidence interval [CI], 1.047–4.316; *P* = .001; Fig. 1).

The causes of death mainly resulted from severe infections (5 cases) and chemotoxicity (2 cases). The clinical problems of patients with DS and ALL were common, with most having associated diseases, secondary immunodeficiencies, and higher toxicities induced by chemotherapy, which explained the high TRM (50%) and the significant difference from those without DS (odds ratio, 1.7; 95% CI, 1.005–2.88; *P* < .0001).

During chemotherapy in the DS-ALL patients, 20 moderate and severe infectious episodes were observed. These infections were bacterial (*Enterococcus*, *Escherichia coli*, *Staphylococcus aureus*, *Klebsiella pneumoniae*), viral (severe varicella, herpes simplex), and fungal (*Aspergillus fumigatus*, *Candida albicans*) in nature.

No prophylactic antibiotic, antifungal, or antiviral protocol was administered. Intravenous immunoglobulin (Ig) supplementation was performed if IgG was <400 mg/dL.

Most infectious episodes (n = 10) occurred during induction therapy: 1 case of sepsis with *Enterococcus*, 2 cases of skin infection (*S aureus*), 1 case of urinary tract infection (*E coli*), 2 cases of bronchopneumonia, 2 cases of digestive infection, 1 case of fungal infection (*C albicans*), and 1 case of herpes.

During the consolidation phase of chemotherapy, 4 infectious episodes were diagnosed as follows: sepsis in 2 cases (*K pneumoniae*, *S aureus*), central venous catheter infection in 1 case, and digestive infection (*Salmonella*) in 1 case. During the reinduction phase, there were 2 cases of fungal infection (*C albicans*, *A fumigatus*), 1 case of viral infection (severe varicella), 1 case of central venous catheter infection, and 1 case of digestive infection. Additionally, in relation to an episode of severe neutropenia during the maintenance phase, 1 patient developed sepsis due to *S aureus.*

Furthermore, other complications related to the toxicity of the chemotherapy are also important to note. We had cases of pancreatitis (n = 2), severe liver failure (n = 1), thrombosis (n = 1), and diabetes (n = 1) after treatment with asparaginase. Severe mucositis, which is a consequence of the increased sensitivity to methotrexate among children with DS,^[17,30,31]^ occurred in 9 patients (64.28%).

Chemotherapy-induced toxicity was higher for methotrexate and L-asparaginase than for other chemotherapeutic agents. The 2 deaths that occurred in induction were due to L-asparaginase toxicity, which resulted in severe liver and multiple organ failure. Two patients had significant reductions in their methotrexate doses to 500 mg/m^2^ and an improvement in their toxicity profiles was observed. Two of the patients were not treated with anthracyclines, but in those that were, no significant increase in cardiotoxicity was observed.

Four patients (28.57%) relapsed, of which 3 were early relapses and 1 was a late relapse. Most patients had bone marrow relapses (75%), but none underwent bone marrow transplantation. Table 3 highlights the biological characteristics of patients who died of DS-ALL, showing the statistically significant correlation of leukocyte counts at the time of diagnosis with survival, but surprisingly, the median leukocyte count was higher in patients who survived (*P* = .026). In the case of non-DS-ALL patients, a statistically significant correlation was maintained between the number of leukocytes at the time of diagnosis and survival, but conversely, patients who died had a higher leukocyte number at onset (*P* = .002)

**Table 3:** Biological features of patients who died with DS-ALL versus non-DS-ALL.

DS-ALL	Died (n = 9)	Alive (n = 5)	*P*
Median WBC count	3600/mm^3^	28,200/mm^3^	.026
Median Hb concentration	9 g/dL	6 g/dL	.118
Median Plt count	46,000/mm^3^	25,000/mm^3^	.406
Median proportion blasts in PB	27%	71%	.125

Non-DS-ALL	Died (n = 40)	Alive (125)	*P*
Median WBC count	24,900/mm^3^	11,300/mm^3^	.002
Median Hb concentration	6.9 g/dL	6.7 g/dL	.404
Median Plt count	45,000/mm^3^	34,000/mm^3^	.755
Median proportion blasts in PB	70%	31%	.074

DS-ALL = Down syndrome with acute lymphoblastic leukemia, Hb = hemoglobin, PB = peripheral blood; Plt = platelet, WBC = white blood cell.

### 3.2 DS-AML patients versus AML patients

In the group of patients with DS-AML, the age at the time of diagnosis was lower (2.5 years) than that in the AML group (11.2 years) (*P* = .151) (Table 4). The female:male ratio was again predominantly female for DS-AML, but this time this feature was observed in both groups of patients, (DS-AML vs AML patients, *P* = .342). The median WBC count at diagnosis was 93,040/mm^3^ (1480–157,250), which was higher than that observed in the DS-ALL group. The median hemogoblin level was 7.7 g/dL (3.3–15.5 g/dL) and the median platelet count was 44,000/mm^3^ (8000–93,000). Regarding the percentages of myeloblasts, the median value in the peripheral blood was 66% and in the bone marrow, it was 80%.

**Table 4:** Clinical and biological features of patients with AML versus DS-AML.

	AML without DS (n = 50)	DS-AML (n = 7)	*P*
Median age at diagnosis, yrs	11.2	2.5	.151
Sex			.342
Male	24 (48%)	2 (28.5%)	
Female	26 (52%)	5 (71.4%)	
Median WBC count (range)	21,500/mm^3^ (1480–593,260/mm^3^)	93,040/mm^3^ (1480–157,520)	.834
Median Hb concentration (range)	7.35 g/dL	7.7 g/dL (3.3–15.5)	.155
Median Plt count (range)	27,000/mm^3^ (2000–261,000)	44,000/mm^3^ (8000–93,000)	.619
CNS infiltration			
Yes	4 (8%)	0 (0%)	
No	46 (82%)	7 (100%)	
Molecular abnormalities	11 (22%)	None	.067

AML = acute myeloid leukemia, CNS = central nervous system, DS = Down syndrome, DS-AML = Down syndrome with acute myeloid leukemia, Hb = hemoglobin, Plt = platelet, WBC = white blood cell.

Based on The French American British classification classifications, in the DS-AML group, 4 patients were diagnosed with AMKL, 1 with AML-M5, 1 with AML-M0, and 1 with AML-M2. There was a statistically significant correlation (*P* = .002) between DS-AML and the morphological type AMKL.

In the DS-AML group, only one child had an associated congenital heart disease (ventricular septal defect and interventricular septal aneurysm). Two patients had a history of low Apgar scores at birth. None of the patients had leukemic infiltrations into the CNS at onset. A girl diagnosed with AMKL at the age of 1 year and 11 months had a particular onset, with thrombocytopenia developing in the previous 6 months concomitantly with progressive bilateral ocular protrusion.

Cytogenetic examination revealed only trisomy 21 in 6 patients, while 1 patient presented with trisomy 21, trisomy 11, the inversion of chromosome 9, and duplication of the long arm of chromosome 1. No patient had any molecular abnormalities identified in the RT-PCR analyses for NPM1, FLT3-ITD, PML-RARα, CBFβ-MYH11, and AML1-ETO.

**Figure 2:**
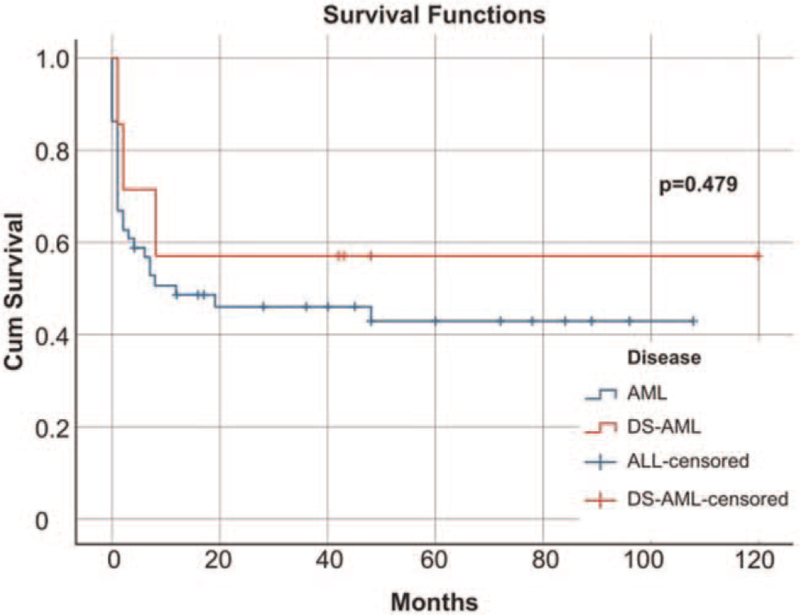
Overall survival in AML without DS (blue line) and DS-AML (red line). AML = acute myeloid leukemia, DS = Down syndrome, DS-AML = Down syndrome-acute myeloid leukemia.

Compared with those with DS-ALL, the patients with DS-AML experienced better outcomes, with an OS of 57.1%. The OS of DS-AML was also better than that of patients with AML without DS, 57.1% versus 45.1% (*P* = .479) (Fig. 2).

Table 5 shows comparative data on outcomes, responses to treatment, TRM, and OS of patients with DS-AML and AML without DS.

**Table 5:** Outcome of patients with DS-AML versus those with AML without DS.

	AML without DS (n = 50)	DS-AML (n = 7)	*P*
Response after induction	25 (50%)	5 (71.4%)	.507
CR	19 (38%)	3 (42.8%)	
PR	6 (12%)	2 (28.5%)	
Early deaths	15 (30%)	2 (28.5%)	
Relapse	6 (12%)	2 (28.5%)	.049
Early relapse	3	2	
Late relapse	3	0	
Bone marrow relapse	5	1	
CNS relapse	0	0	
Extramedullary	1	1	
Treatment-related mortality	8 (16%)	1 (14.2%)	.261
Overall survival	45.1%	57.11%	.479

AML = acute myeloid leukemia, CNS = central nervous system, CR = complete remission, DS = Down syndrome, DS-AML = Down syndrome with acute myeloid leukemia, PR = partial remission.

The relapse rate was 28.50% in the DS-AML group, all of which were early relapses and 12% in the group of patients without DS (*P* = .049). There is a high percentage of deaths (30%) during the 2 induction courses in the AML group.

We noted an extramedullary relapse at the level of the right lower eyelid in the case of a girl with AMKL; the relapse was confirmed by a local biopsy and immunohistochemistry. No patient with DS-AML underwent bone marrow transplantation, and 7 patients (14%) underwent bone marrow transplantation in the AML group.

The complications experienced after chemotherapy in the DS-AML patients were mainly mucositis, skin infections, and hemorrhagic manifestations. TRM is lower (14.2%) in this group than in the DS-ALL group (50%) and similar to that seen in AML patients without DS. Deaths were caused by disease progression (66.66%) and upper gastrointestinal hemorrhages (33.33%). One of the 2 infants under 3 months of age presented with progressive disease and died 14 days after the diagnosis. One of the patients was diagnosed with AML M0 in the adult center at the age of 32 years and 9 months; he died of disease progression after a very early relapse. This patient was treated with the BFM AML 2004 protocol that was also used in the pediatric centers.

## 4 Discussion

Few multicenter studies in developing countries analyzed the outcome and survival of patients with DS and acute leukemia and compared them with those of patients with acute leukemia without DS diagnosed in the same period of time. This retrospective study includes patients from 3 main hemato-oncological centers in Romania.

The diagnosis of acute leukemia patients with DS occurred predominantly in patients below 18 years of age, but there were 2 cases, 1 of DS-ALL and the other of DS-AML, that were diagnosed in adulthood. One patient was followed in 2 centers, first in the pediatric center where he was diagnosed and then in the adult center after reaching the age of 18 years. One of the novelties of this study is the fact that the group of patients with DS and acute leukemia included patients diagnosed or in follow-up in adulthood. The outcome of the 3 patients followed in adulthood was unfavorable. Only one of them survived and it is noteworthy that all 3 patients had relapses; 1 of them had 3 successive bone marrow relapses and underwent 3 lines of treatment until death.

We noted that TRM in the patients with DS-ALL was high and occurred in all phases of therapy (including maintenance), both in the first line protocol as well as in the second-line protocol after relapse and the difference is significant when compared with the group of patients with ALL without DS (*P* < .001). This confirms the data available in the literature and in the study by a group from Ponte di Legno.^[17]^

Of the deaths that occurred in the DS-ALL patients, only 22.2% (2 cases) were because of disease progression. One patient had 3 bone marrow relapses; in that case, the disease was refractory to the second-line protocol LAL REC 2003 and to clofarabine treatment. The other patient who experienced significant dose reductions of methotrexate in the first-line protocol, did not receive anthracyclines, and only one block of cytarabine treatment was administered due to severe anaphylactic shock after administration. Bone marrow relapse occurred during maintenance therapy and the family refused the second-line treatment. None of the patients with DS and acute leukemia underwent bone marrow transplantation since a complete remission could not be obtained after relapse or due to the multiple toxicities encountered during the therapy. Analysis of the biological profile of patients who died of DS-ALL surprisingly showed that death correlated with a lower WBC count at the time of diagnosis, relative to that in living patients. This could be related to the high treatment mortality for DS-ALL patients, with deaths being caused less by disease progression and more by treatment toxicity.

The treatment of patients with DS-ALL is challenging, as it is difficult to find a balance between the doses of chemotherapy necessary to obtain complete long-term remission and those that are highly toxic; individual differences in drug responses complicate matters further. We consider it opportune control of infections with the prophylactic administration of Igs, as well as antibiotics in these patients.^[14]^ Another way to improve the outcome in this subgroup of patients, in the future, will be to use a differentiated therapy depending on MRD. Unfortunately, our study only had 4 patients with acute leukemia and DS who underwent MRD after induction chemotherapy, making it impossible to perform an analysis based on this variable.

The observation that immunotherapy resulted in lower infectious toxicity has led to the question of whether outcomes could be improved by decreasing TRM in patients with DS who have high rates of infection following treatment with conventional chemotherapy regimens. The latest first-line Children's Oncology Group protocol for standard risk B-cell precursor ALL, AALL1731,^[32]^ randomizes patients by introducing 2 cycles of blinatumomab treatment after consolidation, whereas only conventional chemotherapy is used in the other arm. DS-ALL patients are also eligible for treatment with this protocol, but trial results are not yet available.^[32]^

Chimeric antigen receptor (CAR)-T cells are also an attractive option for treating children with DS-ALL, and this category of patients is eligible for studies that assess the efficacy and safety of CAR-T cells (NCT02435849 trial), with the first published data indicating that the efficacy and toxicity profiles do not differ from those of ALL children without DS.^[33,34]^

Usually, children with DS and ALL are excluded from phase 1 and 2 studies with antileukemic agents based on the eligibility criteria (this is justified by the severe side effects observed in this group of patients). However, at this time, it was considered important to conduct clinical trials to introduce immunotherapy to treat children with DS-ALL.^[33]^

There is also an ongoing study, ASIA Down Syndrome Acute Lymphoblastic Leukemia 2016 (NCT03286634), that is currently evaluating the outcomes of patients with DS-ALL using a protocol with lower doses of methotrexate (500 mg/m^2^) and prednisolone, as well as personalized therapy according to the level of the minimum residual disease evaluated by flow cytometry.^[35]^

The better outcome of patients with DS-AML described in the literature^[36]^ was also confirmed in our study. The OS was 57.1%, higher than that of patients with DS-ALL (35.7%, *P* = .438) and that of AML patients without DS which were diagnosed in the same period of time in “Sf Maria” Children's Hospital, 45.1% (*P* = .479). Their TRM was lower than that of the DS-ALL patients (14.2% vs 50%, respectively). Table 6 presents an analysis in parallel of DS-ALL and DS-AML patients regarding the response after induction, relapse rate, survival, TRM, complications after chemotherapy, and cause of deaths. There were no statistically significant differences in terms of response after induction, and the relapse rate was identical in the 2 groups. Obviously, the complications after chemotherapy were more numerous and more severe in DS-ALL patients, which led to treatment delays, dose reductions (5 patients), or even discontinuation of a type of cytostatic in a patient who experienced anaphylactic shock (Cytarabine).

**Table 6:** Outcome of patients with DS and acute leukemia (DS-ALL and DS-AML).

	DS-ALL (n = 14)	DS-AML (n = 7)	*P*
Response after induction	12 (85.7%)	5 (71.4%)	.263
CR	11 (78.5%)	3 (42.8%)	.221
PR	1 (71.4%)	2 (28.5)	.457
Early deaths	2 (14.2%)	2 (28.5%)	
Poor prednisone response	3 (21.4%)	NA	
Relapse	4 (28.5%)	2 (28.5%)	
Early relapse	3 (21.4%)	2 (28.5%)	
Late relapse	1 (7.1%)	0 (0%)	
Bone marrow relapse	3 (21.4%)	1 (14.2%)	
CNS relapse	1 (7.1%)	0 (0%)	
Extramedullary	0 (0%)	1 (lower eyelid) (14.2%)	
Survival	35.7%	57.1%	.438
TRM	7 (50%)	1 (14.2%)	.209
Complication chemotherapy			
Hemorrhagic manifestation	1 (7.1%)	2 (28.5%)	
Skin infections	2 (14.2%)	2 (28.5%)	
Sepsis	4 (28.57%)	1 (14.2%)	
Catheter infections	2 (14.2%)	1 (14.2%)	
Mucositis	9 (64.2%)	3 (42.8%)	
Pancreatitis	2 (14.2%)	0 (0%)	
Diabetes	1 (7.1%)	0 (0%)	
Thrombosis	1 (7.1%)	0 (0%)	
Liver failure	1 (7.1%)	0 (0%)	
Aspergillosis	1 (7.1%)	0 (0%)	
Myopathy	3 (21.4%)	0 (0%)	
Dose reductions			
MTX	2 (14.2%)	0 (0%)	
ARA-C	1 (7.1%)	1 (14.2%)	
Anthracycline	2 (14.2%)	0 (0%)	
Number of deaths by cause	n = 9 (64.2%)	n = 3 (42.8%)	
Disease progression	2 (14.2%)	2 (28.5%)	
Severe bleeding	0 (0%)	1 (14.2%)	
Severe infection	5 (35.7%)	0 (0%)	
Chemotherapy toxicity	2 (14.2%)	0 (0%)	
Median follow-up time	3 years (2 months–6.5 years)	3.5 years (14 days–10.5 years)	

ARA-C = cytosine arabinoside (cytarabine), CNS = central nervous system, CR = complete remission, DS-ALL = Down syndrome with acute lymphoblastic leukemia, DS-AML = Down syndrome with acute myeloid leukemia, MTX = methotrexate, PR = partial remission, TRM = treatment related mortality.

The only statistically significant difference between the DS-AML group and the DS-ALL group was regarding the number of leukocytes at the time of diagnosis, which was much higher in patients with DS-AML (*P* = .018).

HD-ARA-C cycles were effective in treating extramedullary relapse at the level of the lower eyelid in the case of a girl with DS-AMKL who is currently in complete remission. This confirms the results reported in ex vivo studies that have shown an increased sensitivity to cytarabine in the myeloblasts of DS-AML patients.^[8–11]^ HD-ARA-C cycles have been used in our patients, either in the first-line protocol, in consolidation, or in the treatment of relapse. The Children's Oncology Group AAML 0431 trial showed that the use of HD-ARA-C early in the first-line protocol (i.e., in the second course of induction), improves the survival and response rate in children with DS-AML.^[37]^

As with DS-ALL, there are currently no published results of clinical trials using new targeted therapies in those with DS-AML. To date, only single case reports describing treatment with demethylating agents or histone deacetylase inhibitors have been published in patients with DS and refractory or relapsed AML.^[38,39]^

The main limitation of our study is that it included a relatively small number of patients with acute leukemia and DS. This is due to the low proportion of children with DS and acute leukemia relative to the proportion of cases of acute leukemia (2.42% children diagnosed with ALL in “Sf Maria” Children's Hospital between 2009 and 2018 were DS-ALL).

Another limitation of the study is that genetic tests such as *GATA1* mutation for DS-AML or *CRLF2* mutation for DS-ALL could not be performed.

Of relevance is our experience in the treatment of DS-ALL patients where due to the fragility of the host, were encountered high toxicities of chemotherapy, high rates of infections, and significant differences in survival between the non-DS-ALL and DS-ALL children (Fig. 1).

In conclusion, while the OS of pediatric patients with ALL has continuously improved in recent years in our country, the outcome remained significantly unfavorable for patients with DS-ALL, for whom both relapse rate (28.5% vs 13.3%, *P* = .13) and TRM (*P* < .0001) are higher. The outcome of this subgroup of patients can be improved by the establishment of specific DS-ALL protocols, strict differentiation of therapy according to MRD, and observation of a strict prophylaxis of infections. Regarding DS-AML patients, we had a better experience: they had a better OS, lower TRM, a good response to HD-ARA-C cycles, and a better outcome than did patients without DS.

We believe that presenting real-life clinical experience of centers from a country with less economic resources can be helpful for those facing similar problems. We believe that due to the particularities of the host, DS-ALL and DS-AML should be considered as independent diseases and should have specific treatment protocols.

## Acknowledgments

The authors would like to thank Editage (www.editage.com) for English language editing.

## Author contributions

**Conceptualization:** Madalina Petronela Schmidt, Daniel Coriu.

**Data curation:** Madalina Petronela Schmidt, Anca Colita, Anca Viorica Ivanov.

**Formal analysis:** Madalina Petronela Schmidt.

**Investigation:** Anca Colita.

**Methodology:** Madalina Petronela Schmidt.

**Resources:** Madalina Petronela Schmidt, Anca Colita, Anca Viorica Ivanov.

**Software:** Anca Viorica Ivanov.

**Supervision:** Daniel Coriu, Ingrith Crenguta Miron.

**Validation:** Daniel Coriu, Ingrith Crenguta Miron.

**Visualization:** Daniel Coriu.

**Writing – original draft:** Madalina Petronela Schmidt.

**Writing – review & editing:** Madalina Petronela Schmidt.
